# Collective behavior as a driver of critical transitions in migratory populations

**DOI:** 10.1186/s40462-016-0083-8

**Published:** 2016-07-15

**Authors:** Andrew Berdahl, Anieke van Leeuwen, Simon A. Levin, Colin J. Torney

**Affiliations:** Santa Fe Institute, 1399 Hyde Park Rd, Santa Fe, 87501 NM USA; Department of Ecology & Evolutionary Biology, Princeton University, Princeton, 08544 NJ USA; Centre for Mathematics and the Environment, University of Exeter, Penryn Campus, Cornwall, UK

**Keywords:** Collective navigation, Migration, Population collapse, Dispersal, Local adaptation, Anadromous fish, Migratory ungulates, Migratory birds, Migratory marine fish

## Abstract

**Background:**

Mass migrations are among the most striking examples of animal movement in the natural world. Such migrations are major drivers of ecosystem processes and strongly influence the survival and fecundity of individuals. For migratory animals, a formidable challenge is to find their way over long distances and through complex, dynamic environments. However, recent theoretical and empirical work suggests that by traveling in groups, individuals are able to overcome these challenges and increase their ability to navigate. Here we use models to explore the implications of collective navigation on migratory, and population, dynamics, for both breeding migrations (to-and-fro migrations between distinct, fixed, end-points) and feeding migrations (loop migrations that track favorable conditions).

**Results:**

We show that while collective navigation does improve a population’s ability to migrate accurately, it can lead to Allee effects, causing the sudden collapse of populations if numbers fall below a critical threshold. In some scenarios, hysteresis prevents the migration from recovering even after the cause of the collapse has been removed. In collectively navigating populations that are locally adapted to specific breeding sites, a slight increase in mortality can cause a collapse of genetic population structure, rather than population size, making it more difficult to detect and prevent.

**Conclusions:**

Despite the large interest in collective behavior and its ubiquity in many migratory species, there is a notable lack of studies considering the implications of social navigation on the ecological dynamics of migratory species. Here we highlight the potential for a previously overlooked Allee effect in socially migrating species that may be important for conservation and management of such species.

**Electronic supplementary material:**

The online version of this article (doi:10.1186/s40462-016-0083-8) contains supplementary material, which is available to authorized users.

## Background

Across the globe animals make long-distance migrations to access the resources needed for survival and reproduction. Migration allows organisms to take advantage of ephemeral resources, avoid harsh conditions, minimize predation, and rear their offspring in suitable environments. To fuel long distance travel, migratory species are major resource consumers, while their energy stores also make them attractive prey items, thus they are important at multiple levels in food webs. At the broadest scale, migrations shape ecosystems by altering trophic interactions, generate fluxes of limiting nutrients, and determine connectivity between local populations and communities, both by linking community dynamics across space, and by structuring population genetic patterns [1–4].

Many animal migrations involve impressive feats of navigation. For example, Pacific salmon are able to find their way over thousands of kilometers to their precise natal site, while the blue wildebeest tracks shallow and noisy environmental gradients to follow favorable feeding conditions. To navigate along migratory routes animals are thought to employ celestial and geomagnetic cues, environmental and resource gradients (temperature, salinity, odor etc.) as well as land marks (see [5] for a review). Recent theoretical [6–9] and empirical [10–16] work suggests that by traveling in groups animals may increase their ability to find their way. Indeed across many taxa, migrations are undertaken by large social groups [17] and in some species migratory distance is positively correlated with group size [18].

There are three general ways in which migratory animals might benefit from collective navigation. First, by spanning a larger spatial scale, groups are thought to act as distributed sensory arrays [19]. In this scenario taxis emerges at the group level from a combination of social and environmental interactions, even when individuals have weak or no gradient sensing ability [6, 10]. Second, by flocking or schooling, groups average out directional preferences of the constituent individuals. This averaging over many independent estimates, known as the *many wrongs* principle, tends to prune out errant guesses, and if there is no overall bias, to hone in on the correct direction for large group sizes [7, 20–23]. Finally, traveling as a group allows for social learning [12, 24–29] and collective memory [8] of migratory routes.

Empirical evidence suggests animals do benefit from these mechanisms in nature: anadromous salmon migrate back to their home streams more accurately in years of greater abundance [11, 30]; flocks of pigeons can home more accurately than individuals [13, 16]; schools of pelagic marine larvae can orient more accurately than individuals [14]; migratory vultures born nearer to dense migration corridors are more likely to successfully navigate [31]; and movement data from individual wildebeests suggest that these ungulates move along gradients thought to be too shallow to be measured by an individual [32].

Here we explore the potential impact of collective navigation on the population, and movement, dynamics of migratory species. In recent decades there has been much focus on the idea that ecological systems undergo abrupt transitions between alternate stable states [33–35]. Several models have been proposed to further investigate this phenomenon, including multi-species models of community dynamics [36], two species models of predator-prey or host-parasite interactions [37], and single species models exhibiting strong Allee effects [38]. Common to all these models is the presence of bistability or multistability in the underlying dynamical system. Rapid transitions between attractors may occur due to a parameter change causing a bifurcation in the deterministic system [39], or through stochastic fluctuations causing the system to flip from one state to another [40].

For single species models, there is bistability when the population displays a strong Allee effect. Fundamentally this occurs when the growth rate of the population is negative below a critical threshold. Various mechanisms may lead to Allee effects and ecological models have revealed that mate finding [41], cooperative breeding [42] and the use of social information during habitat selection [43] are potential drivers of these effects. In this work we propose that collective navigation is a further, unexplored mechanism that can cause migratory populations to collapse. If migrants improve the accuracy of their movement decisions as a result of collective behavior then fragmentation or reduction of populations could impact migration ability. Further, if large populations are sustained by successful migrations, a reduction in navigational accuracy would cause additional reduction of population size. Through a model-based analysis of two common forms of migration we reveal that these processes result in a positive feedback mechanism that may cause abrupt changes in abundance or movement patterns of collectively migrating species.

## Methods

We define migration as the cyclic relocation of individuals over a larger spatial scale than normal ranging or station-keeping movements, with individual trajectories characterised by increased persistence in heading and a suppression of responses to local stimuli [44, 45]. We consider two generalized types of migrations within this definition: i) breeding migrations, in which individuals travel to discrete fixed end-points to reproduce, typical of anadromous fish and many species of migratory birds; and ii) feeding migrations, characterized by continual motion that tracks regions of favorable conditions, common in marine fish and terrestrial mammals, particularly ungulates.

In this section we describe the three models we have developed of collective migration. The first model considers a breeding migration of semelparous organisms. The second model extends this breeding migration model to a metapopulation distributed over breeding sites. The final model captures feeding migrations involving continuous movement that tracks a resource field. In all models we assume females determine the dynamics (males are not limiting) and do not explicitly consider separate sexes. Definitions of all model parameters and their units are shown in Table 1.

**Table 1:** Parameter values used for simulations

Symbol	Description	Units	Value		
			Breeding single site	Breeding multi site	Feeding
*δ*	Growth rate of resource	1/time	0.1	0.1	–
*R*_*max*_	Carrying capacity of resource	# _*R*_	1	1	–
*α*	Uptake rate of resource by consumer	# _*R*_/(# _*N*_·time)	5×10^−5^	5×10^−5^	–
*b*_*m*_	Maximum birth rate	Unitless	5	5	–
*D*	Half-max consumption/birth rate level of *R*	# _*R*_	0.5	0.5	–
*τ*	Maturation time	Time	1	1	–
*p*	Plasticity relative to environmental heterogeneity	Probability	–	0≤*p*≤1	–
*a*_*o*_	Individual homing accuracy	Probability	0≤*a*_*o*_≤1	0	0
*C*	Half-max accuracy level of *N*	# _*N*_	1000	1000	2.5
*h*	Additional mortality due to harvesting or migration impedance	Probability	0≤*h*≤1	0≤*h*≤1	0≤*h*≤*r*
*r*	Maximum growth rate of migratory consumer	1/time	–	–	0.01
*K*_*max*_	Peak carrying capacity	# _*N*_	–	–	10
*K*_*o*_	Background carrying capacity	# _*N*_	–	–	1
*σ*	Width of wave of favorable conditions	Distance	–	–	1
*v*_*K*_	Speed of wave of favorable conditions	Distance/time	–	–	0.1
*v*_*m*_	Maximum speed of migratory consumers	Distance/time	–	–	0.17

### Breeding migrations

Many organisms migrate back to fixed, discrete natal sites to breed. This behavior is common across many taxa including fish, birds, mammals and insects [17]. Anadromous salmon provide an archetypal example; after spending years feeding in the rich marine environment these fish travel vast distances up rivers, often returning to the precise point at which they were born [46, 47]. Strong local adaptation to certain conditions means that failing to navigate back to that specific site can result in a significant reduction in fitness [48–50]. Similarly, migratory birds travel annually from southern wintering grounds to discrete sites in temperate or Arctic regions to breed [51]. Empirical evidence suggests that by traveling in groups animals on breeding migrations might home more accurately to their natal site [11, 12, 14, 31].

The following two models describe the dynamics of a population reliant on breeding migrations. These models are inspired by the life history of anadromous salmonids. Due to collective navigation, individuals navigate more accurately to their natal site when at higher densities. We begin by considering the dynamics of a single breeding population where an error in navigation results in zero fecundity. We then extend this model to a metapopulation of *m* discrete breeding sites and relax the zero fecundity assumption for straying individuals by introducing a local adaptation parameter.

#### Model 1: Breeding migration with single natal site

We consider a population of semelparous organisms, which, as juveniles, migrate from a natal site to a feeding site and then as adults return to their natal site to breed and then perish. Consumer population size is denoted *N*. We assume that the resource density, *R*, on the feeding grounds follows semi-chemostat growth in absence of grazing (this assumes constant resource productivity with turnover rate *δ*, and maximum resource density *R*_*max*_). Feeding by consumers is assumed to follow a monotonically increasing, but saturating, function of the resource abundance (type II functional response), resulting in the resource dynamics being defined as follows:

1 $$ \frac{dR}{dt} = \delta(R_{max} - R) - \alpha N \left(\frac{R}{D+R}\right),   $$

where *α* scales the uptake rate of the resource by the consumer. Similarly, the per capita fecundity of the consumers, *b*, is proportional to their uptake rate of the resource,

2 $$ b(R) = b_{m}\left(\frac{R}{D+R} \right),   $$

where *b*_*m*_ is the maximum fecundity and *D* is a parameter controlling the non-linearity of the term in parentheses (commonly called the half-saturation coefficient). With this formulation of consumer fecundity, we reflect the assumption that resource density on the feeding grounds determines a component of survival and reproductive capacity. At the same time, we explicitly assume that the system is open, since the component of the life cycle taking place off the feeding grounds is not incorporated in terms of a resource interaction. We include an additional mortality term, *h*, reflecting mortality occurring before or during the adult migration, due to harvesting or blockage of the migration route. Finally, to account for collective navigation, we suppose that the fraction of individuals successfully completing the migration, *a*, is an increasing function of *U*, the number of individuals attempting the migration and who also survive the additional mortality,

3 $$ a(U) = a_{0} + (1-a_{0})\left(\frac{U}{C+U} \right).   $$

Here *a*_0_ gives the accuracy of a lone traveler and *C* parametrizes how swiftly accuracy increases with abundance. We assume that accuracy is a monotonically increasing function that begins at *a*_0_ and saturates at 1. This functional form of Eq. (3) matches the general trend found in empirical studies of salmon homing as a function of run density [11, 30], however we stress that our results are not dependent on the exact form of this equation. On the breeding grounds, each adult gives birth to *b*(*R*) (Eq. (2)) juveniles and then dies. The dynamics of the migratory population in continuous time are given by

4 $$ \frac{dN}{dt} = b(R)a(U)U - \frac{1}{\tau}N,   $$

where $U=(1-h)\frac {1}{\tau }N$ (the number of individuals attempting the migration and surviving the additional mortality, *h*) and *τ* is the characteristic time to maturation (note *h*≤1, *τ*≥1).

#### Model 2: Breeding migration with multiple natal sites

We extend the model formulation by considering the possibility of multiple natal sites. Now individuals failing to successfully navigate to their natal site end up at some other site. We assume that due to local adaptation individuals straying from the site to which they are adapted will have reduced fitness (given by decreasing the fecundity by a factor *p*, where 0≤*p*≤1) with respect to their homing conspecifics. (Note that the factor *p* could also include the probability of not reaching any site at all.) If we assume symmetry between the natal sites, we need only consider two populations, the number of locally adapted, homing individuals, *N*_*h*_, and the number of non-locally adapted individuals (be they strayers or individuals homing to a natal site to which their parent was not adapted), *N*_*s*_, at a given site. For *m* natal sites the dynamics of the migratory population are given by

5 $$ {{}\begin{aligned} \frac{{dN}_{h}}{dt} = b(R)(1-h) \left\{ a(U)\frac{1}{\tau}N_{h} + \frac{1}{m}\left(1-a(U)\right)\frac{1}{\tau}N_{s} \right\} - \frac{1}{\tau}N_{h} \end{aligned}}  $$

6 $$ {{}\begin{aligned} \frac{{dN}_{s}}{dt} = p b(R)(1-h)\left\{ a(U)\frac{1}{\tau}N_{s} + \frac{m-1}{m}\left(1-a(U)\right)\frac{1}{\tau}(N_{h}+N_{s})\right\} - \frac{1}{\tau}N_{s}, \end{aligned}}  $$

where the total number of individuals attempting migration, and surviving mortality at a single site is now $U=(1-h)\frac {1}{\tau }(N_{h} + N_{s})$. If the number of sites is large (*m*>>1) we may neglect the offspring of strayers that return back to the site for which they are adapted by chance and the equations simplify to,

7 $$\begin{array}{*{20}l} \frac{{dN}_{h}}{dt} \!&= b(R)(1-h)\frac{1}{\tau}\Bigg\{a(U)N_{h} \Bigg\} - \frac{1}{\tau}N_{h}  \end{array} $$

8 $$\begin{array}{*{20}l} \frac{dN_{s}}{dt} \!&= p b(R)(1\,-\,h)\frac{1}{\tau}\Bigg\{ N_{s} \,+\, \left(1\,-\,a(U)\right)N_{h}\Bigg\} \!- \frac{1}{\tau}N_{s}.  \end{array} $$

Resources on the feeding grounds are again given by

9 $$ \frac{dR}{dt} = \delta(R_{max} - R) - \alpha (N_{h} + N_{s}) \frac{R}{D+R}.   $$

### Feeding migrations

Feeding migrations are characterized by the tracking of favorable conditions for foraging. When resources vary periodically according to seasonal climate, these migrations tend to be cyclic loops [52] and this form of migration appears to be the dominant form of terrestrial migration [53] as well as being common in marine environments [54]. On land, responding to spatial or temporal cues that indicate (or predict) resource quality can keep animals in prime feeding conditions while leading them on an annual loop migration. Wildebeest of the Serengeti provide a classic example, traveling an annual 650 km loop while tracking regions of grass height, greenness and new growth [55–57]. Gazelles also seemingly following gradients of grass quality [58], but in more stochastic environments, make nomadic style movements rather than following a specific annual path [59, 60].

In this section, we model the dynamics of a population migrating to track a variable resource on an annual time-scale. We note that depending on the nature of the individual behavior and response to environmental cues, the movements may either be migration (involving the inhibition of responses to local resources [57]) or more properly considered long-range foraging movements (direct response to a moving resource [52]). While inspired by the annual loop around the Serengeti made by wildebeest, our model assumes only that animals respond to a cue that indicates where favorable regions will be, and hence can apply equally to any migration or nomadic movement where individuals track resource gradients more effectively at higher densities.

#### Model 3: Feeding migrations

**Fig. 1:**
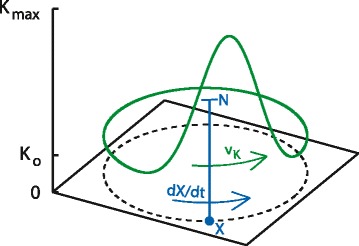
Feeding migration model schematic (Model 3). Space is restricted to a 1 dimonsional loop (*black dashed line*). The green curve shows the time-varying carrying capacity at all points in this space (Eq. 10). A patch of favorable conditions translates steadily through space with speed *v*_*K*_. The location and height of the blue bar indicate the location (*X*) and size (*N*) of the migratory population, respectively. The population tracks the patch with a speed, $\frac {dX}{dt}$, that is dependent on the local strength of the resource gradient and the size of the population (Eq. 13). The *z*-axis applies to both carrying capacity (*green curve*) and population size (height of *blue bar*) and is thus in units of number of animals. See Additional files 2, 3, and 4 (Animation 1–3) for animations of the simulation

We consider a patch of good conditions (rain, vegetation, water temperature, etc.) traveling with constant speed, *v*_*K*_, in an annual loop. Because this patch travels in a loop, we restrict our spatial model to one dimension with periodic boundary conditions. The conditions, as a function of time and space, are given by

10 $$ K(\theta,t) = (K_{max} - K_{o}) e^{\frac{-\phi(\theta,t)^{2}}{2\sigma^{2}}} + K_{o},   $$

where *ϕ*(*θ*,*t*) is the distance between a given position, *θ*, and the location of the center of the patch, *v*_*K*_*t*. More precisely,

11 $$ \phi(\theta,t)=\text{min} \left\{ \begin{array}{l} \text{mod}[(\theta-v_{K} t),L] \\ L - \text{mod}[(\theta-v_{K} t),L], \end{array}\right.  $$

with *L* being the length of the loop (see Fig. 1 for an illustration). Note, that this rather cumbersome expression for *ϕ* simply imposes the periodic boundary conditions. We assume the resource responds to these conditions and the consumer on a fast time scale so we can neglect the resource dynamics and thus have the conditions set the local carrying capacity for the migratory consumer.

In this environment we consider a group of consumers having population size *N* and location *X*. The size of the group follows logistic growth based on the current resource conditions at its location, along with some additional mortality, *h*, (e.g. due to harvesting or blockage of the migration route),

12 $$ \frac{dN}{dt} = rN\left(1-\frac{N}{K(X,t)}\right) - hN.   $$

The group attempts to track the patch of favorable conditions by moving in the direction of the local gradient of the resource field (Eq. (10)). We assume that the speed at which the group travels up the gradient in *K* is proportional to the strength of that gradient at their location and the group’s size-dependent ability to follow this gradient, which is again given by Eq. (3). Thus the movement of the group follows

13 $$ \frac{dX}{dt} = v_{m} \left. \left\langle \frac{\partial K(\theta,t)}{\partial \theta} \right\rangle \right|_{\theta=X} a(N)   $$

where *v*_*m*_ is the maximum speed, *a*(*N*) is the group’s ability to respond to the gradient (Eq. (3)) and the <> denote that the gradient field is normalized by the maximum value of the field, and is thus unitless. (Note that this model can be mapped to a two-dimensional system where the patch of favorable conditions follows an arbitrary (nomadic) path, and *X* is the distance from the group to the center of the resource patch.)

At equilibrium, the population size will be constant $\left (\frac {dN}{dt} = 0 \right)$ and the speed of the migration will match the speed of the resource patch $\left (\frac {dX}{dt} = v_{K} \right)$. Imposing these conditions onto Eqs. (12) & (13) leads to the following, *stationary*, system of equations

14 $$\begin{array}{*{20}l} \bar{N} &= K(\bar{X},0)\left(1 - \frac{h}{r} \right) \end{array} $$

15 $$\begin{array}{*{20}l} \bar{N} &= \frac{\left(v_{K}-a_{o} v_{m} \left\langle \left.\frac{dK}{d\theta}\right\rangle\right\rvert_{\bar{X},0} \right)C}{v_{m} \left\langle \left.\frac{dK}{d\theta}\right\rangle \right\rvert_{\bar{X},0}-v_{K}},  \end{array} $$

which we solve numerically (Fig. 5).

### Numerical methods

**Fig. 2:**
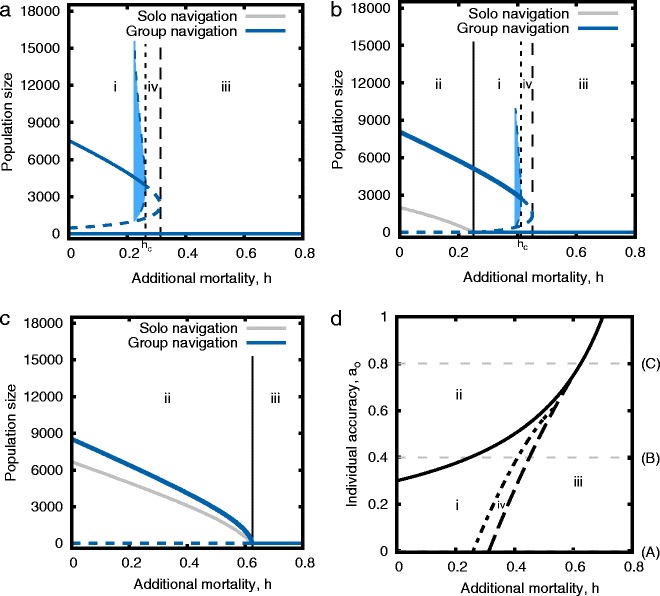
Collapse of breeding migration (Model 1). Panels **a**–**c** show stationary solutions for $\bar {N}$ from Eqs. (1) & (4) as a function of mortality, for individual accuracies of *a*_*o*_=0.0, 0.4 and 0.8 respectively. Results including collective navigation (*a*(*U*) as in Eq. (3)) are *blue* while those with independent navigation (*a*(*U*)=*a*_*o*_) are *grey*. The shaded *blue* regions depict an unstable limit cycle, inside of which the dynamics are locally attracted to the stable equilibrium within, while globally (and in response to extreme perturbations) the system collapses to the stable equilibrium where the population is extinct. *Vertical lines* correspond to the boundaries (bifurcation points) in panel **d**. Note in panel **a** the *grey* curve is not visible because it is equal to zero for all *h*. Panel **d** traces the branching point (*solid line* – Eq. (17)), Hopf point (*dotted line*) and limit point (*dashed line*) through *h*- *a*_*o*_ space. Faint *horizontal lines* correspond to the cross-sections depicted in panels **a**–**c**. The qualitatively distinct states of the system in the different parameter regions are: i) Group navigation is bistable [high *N*_*h*_|*N*_*h*_ extinct], solo navigation is not possible; ii) Both group and solo navigation are possible, group navigating population densities are higher; iii & iv) Neither group nor solo migration can persist

**Fig. 3:**
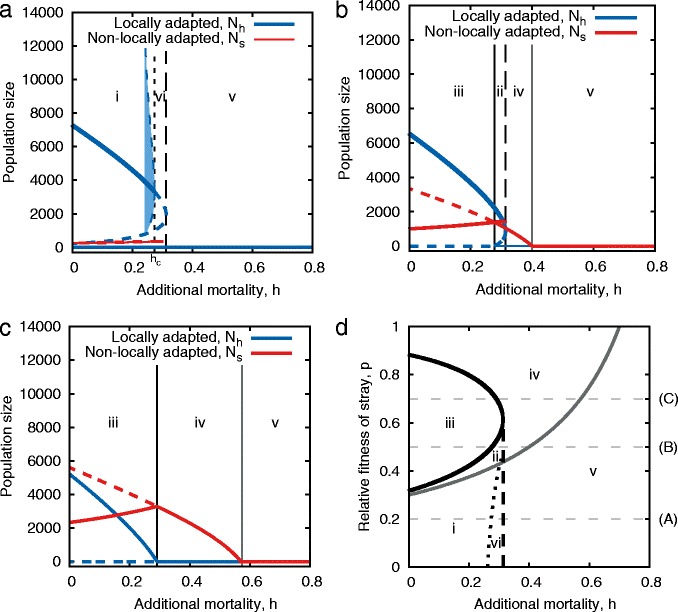
Collapse of population size and structure in multi-site breeding migration (Model 2). Panels **a**–**c** show equilibrium population size from Eqs. (7)–(9) as a function of mortality, for relative fitness of non-locally adapted individuals of *p*=0.2, 0.5 and 0.7 respectively. Locally adapted populations (*N*_*h*_) are *blue* while those for non-locally adapted populations (*N*_*s*_) are *red*. See Fig. 2 legend for details. Panel **d** traces the branching points (*solid lines*), Hopf points (*dotted lines*) and limit points (*dashed lines*) through *h*-*p* space. The *black* and *grey* solid lines correspond to branching points for *N*_*h*_ and *N*_*s*_ respectively. The qualitatively distinct equilibrium states are: i) Bistable [high *N*_*h*_, low *N*_*s*_| both extinct]; ii) Bistable [high *N*_*h*_, low *N*_*s*_|*N*_*h*_ extinct, *N*_*s*_ present]; iii) Bistable ^∗^ [Both *N*_*h*_ and *N*_*s*_ present |*N*_*h*_ extinct, *N*_*s*_ present (^∗^Not stable to invasion by the *N*_*h*_ type, however once out of the system it could take evolutionary time to recover them, so it may be stable on short time-scales.)]; iv) *N*_*h*_ extinct, *N*_*s*_ present; v & vi) Both extinct

Numerical analysis in this paper was performed using Matlab (version R2013a). We used the MatCont package, version 5.4, [61] to perform the bifurcation analyses presented in Figs. 2d & 3d and to find the limits of the oscillatory solutions in Figs. 2a, b & 3a. The equilibrium solutions to differential equations in all other figures were obtained using the Matlab differential equation solver ode45().

## Results

### Results for Model 1: Breeding migration with single natal site

We find the equilibrium states of the model by numerically simulating Eqs. (1) & (4). (See Table 1 for full parameter definition and values used.) Additionally we modify Eq. (4), replacing the collective accuracy term, *a*(*U*), by the individual accuracy, *a*_*o*_, to model the same system without collective effects.

Collective navigation allows populations to exist for ranges of parameter space where populations of individuals navigating independently are not viable (Fig. 2, zone i). Further, populations using collective navigation are always greater in size than populations navigating individually (Fig. 2, zones i & ii) unless both are equal to zero (Fig. 2, zones iii & iv).

When individuals navigate independently (grey curves) the equilibrium population size declines continuously with increasing values of additional mortality, *h*, following

16 $$ \bar{N} = a_{0} b_{m} (1-h) \frac{\delta}{\alpha}\left(R_{max} - \frac{D}{a_{0} b_{m} (1-h) -1} \right).  $$

For low values of *h*, the population with collective navigation also exhibits monotonously declining population size with increasing mortality (Fig. 2a–c). High levels of mortality preclude persistence in both models. However, in the case where we include collective accuracy in the population dynamics, we find an intermediate region of mortality levels where the population dynamics are characterized by the occurrence of alternative stable states (bistability), reflecting an Allee effect. Close to the persistence boundary, small changes in the level of additional mortality, *h*, can invoke drastic, and discontinuous, changes in the population state. In addition to a limit point bifurcation (also known as a fold or saddle-node bifurcation), which connects the two non-zero equilibrium branches, the system exhibits a sub-critical Hopf bifurcation, demarcating a critical mortality level, *h*_*c*_, beyond which the stable, non-zero equilibrium is unstable and the system collapses to the stable zero-equilibrium. The population can also collapse even if *h*<*h*_*c*_ in the case of an external perturbation that brings the population size outside of the basin of attraction of the stable, positive equilibrium existing within the unstable limit cycle (shaded blue region, but also see Additional file 1: Figure S2), or below the unstable equilibrium (dashed blue line in Fig. 2a–c and Additional file 1).

Due to the bistability, a collapse as a result of, for example, over-harvesting, cannot simply be reversed by lowering the mortality level. Since the extinct-state is stable, the population size, *N*, must be brought above the unstable equilibrium (dashed blue line) or within the boundary of attraction of the stable, positive equilibrium exisisting within the unstable limit cycle (blue shaded region and Additional file 1: Figure S2). This hysteresis is present whenever the collectively navigating population is viable but the independently navigating population is not (Fig. 2d, zone i). A threshold value of individual accuracy, *a*_*c*_, below which an individual strategy is not viable and a collective strategy is vulnerable to collapse, defines the upper boundary to this region (solid line in Fig. 2d) and is given by,

17 $$ a_{c} = \frac{D/R_{max}+1}{b_{m}(1-h)}.   $$

The extent of this bistability region depends on the level of individual accuracy, *a*_*o*_ (Fig. 2d).

In Fig. 2 we show the different regions in parameter space with qualitatively distinct dynamics: we plot the equilibrium size of the population, $\bar {N}$, as a function of additional mortality, *h* in panels A–C, while panel D traces the branching (solid line), Hopf (dotted line) and limit (dashed line) points through *h*- *a*_*o*_ space. See Additional file 1 for *N*-*R* phase portraits, depicting the basins of attraction, and the dynamics, around these fixed points.

### Results for model 2: Breeding migration with multiple natal sites

At a general level, the model including multiple breeding sites shows qualitatively similar dynamics in response to varying the mortality level as the single-site model: When increasing *h* starting from low values, the population exhibits a stable equilibrium with monotonically decreasing numbers, whereas at high mortality persistence is precluded (Fig. 3a–c). Again, intermediate *h*-values give rise to a range of bistability. However, there is a greater diversity to the types of bistability, due to the two types of individuals (those locally adapted to the breeding site and those not). As in the single site model the entire population can collapse, but here we can also observe crashes in local adaptation, without necessarily a collapse in population size.

The parameter boundaries delineating regions with qualitatively different equilibrium dynamics are dependent upon *h* as well as *p* (Fig. 3d). When the amount of local adaptation is high ($p \lesssim 0.3$, i.e. strays have 30 % the fecundity of homing individuals) the patterns for the homing population (blue curves) are qualitatively similar to the single site solution shown in Fig. 2a (along with a small population of *N*_*s*_). For *p* values between zero and the intersection of the Hopf and limit points (i.e. the intersection of the dotted and dashed lines in Fig. 3d), we observe a complete crash of the population beyond the Hopf bifurcation, as in the single site model (Fig. 3a).

**Fig. 4:**
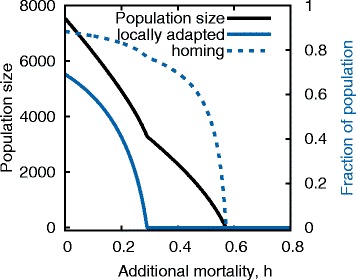
Collapse of local adaptation (Model 2). Equilibrium size of total population, *N*_*h*_+*N*_*s*_, (*black curve*), fraction of the population which is locally adapted, $\frac {N_{h}}{N_{h} + N_{s}}$, (*solid blue curve*) and the fraction of the population which is returning to their natal site (*dashed blue curve*) as a function of additional mortality. Parameters are as in Fig. 3 c. For modest levels of local adaptation (here *p*=0.7) the population size declines nearly linearly as a function of additional mortality. However, the locally adapted fraction of the population crashes dramatically, and non-linearly, at a level of *h* for which the homing rate is still high and the population size seems robust

For values of *p* where the limit point (dashed line) separates zones ii and iv, raising mortality above the limit point causes a discontinuity in, and complete collapse of, the equilibrium level of locally adapted types. However, the non-locally adapted population does not crash to zero under these conditions (Fig. 3b). There is a hysteresis effect such that once the locally adapted population is lost, the non-locally adapted dominated system is stable and the locally adapted type cannot invade. Zone iii has similar properties, except here the equilibrium with $\bar {N}_{h}=0$ is not mathematically stable. Nevertheless, because it might take evolutionary time for locally adapted types to emerge, this state may be considered ecologically stable. At these levels of *p* the boundary between zones iii and iv does not mark a collapse in population size, but one of population genetic structure (Figs. 3c & 4). We note that this collapse in local adaptation could easily be missed if population managers were observing total population size or even rates of straying and dispersal (Fig. 4).

Finally, at very low levels of local adaptation ($p \gtrsim 0.9$) the non-locally adapted phenotype dominates the system and the dynamics of $\bar {N}_{s}$ would be qualitatively similar to the solo-navigation dynamics (i.e. grey curve in Fig. 2c) while $\bar {N}_{h}$ would be zero.

In Fig. 3a–c we plot the equilibrium numbers of locally adapted, $\bar {N}_{h}$, and non-locally adapted, $\bar {N}_{s}$, individuals at a single site. Panel D traces the bifurcation points of the system, in particular, the fold, Hopf and branching points through *h*−*p* space. There are six distinct zones (i-vi) and five qualitatively distinct fixed-*p* cross sections with dynamics as described above.

### Results for Model 3: Feeding migration

**Fig. 5:**
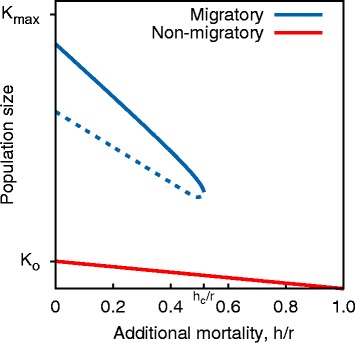
Collapse of feeding migration (Model 3). Numeric solutions for $\bar {N}$ given the dynamics described in Eqs. (12) & (13). For low levels of additional mortality (*x*-axis) the system is bistable, exhibiting either a large population migrating at speed *v*_*K*_ (*blue curve*) or a small, stationary population which relies on the background resource level, *K*_*o*_, with an occasional pulse when the favorable patch passes by (*red curve*). A limit point defines the critical level of *h* above which the migratory population cannot exist

In Fig. 5 we plot the equilibrium population size, $\bar {N}$, from the solution to Eqs. (14) & (15). When additional mortality is low and population size is high (blue curve) the population migrates at speed *v*_*K*_, tracking the traveling wave of good conditions (Eq. (10)). As mortality increases, the group’s size decreases, along with its ability to track the gradient, until it reaches a critical point, *h*_*c*_. At this point the population becomes too small to effectively track, and keep pace with, the patch of good conditions; it is left behind and collapses. The system exhibits hysteresis in both population size and migratory state, so once the collapse has occurred, lowering the mortality to pre-collapse levels is insufficient for the population to recover its size or ability to migrate. The non-migratory solution (red curve) is approximately $\bar {N} = \left (1 -\frac {h}{r} \right) K_{o}$. We note that both the ‘migratory’ and ‘non-migratory’ populations follow the same movement rule (climb the resource gradient with speed given by Eq. (13)), but only the migratory population is large enough to be able to keep pace with the patch of favorable conditions.

## Discussion

This study focuses on the population dynamics of species that utilize collective navigation during their migrations. Theory and experimental evidence suggest that social interactions may help animals traveling in groups to find their way when navigating through challenging environments, and that these effects should increase for larger groups [6, 7, 10–16, 20–23, 62, 63]. The potential effects, from these individual-level mechanisms, at the population or community level are, however, not well studied or described. With the current study we offer insight into the ecological dynamics that may result from individuals in migratory populations traveling in groups. Rather than using individual-based models and assuming specific interaction rules, we bestow individuals in ODE population models with generic group-level benefits, based on empirical studies of collective behavior. We use these models to investigate to what extent group level benefits affect the possible population-level dynamics of social species. We find that while collective behavior can positively affect migratory populations, it can also result in non-linear effects and sudden collapses under broad environmental conditions.

The types of migrations we have studied here represent two of the most prevalent types of migrations: travel to specific breeding sites, and movement to track regions of favorable conditions. Our models were based stylistically on the movement ecology of salmon (Models 1 & 2) and wildebeests (Model 3); however, they should apply in a generic sense to a wide array of taxa. Moreover, the specific choice of population model, outfitted with a collective accuracy term, should not qualitatively change our results. This is because our results are a product of the feedback between migratory ability and population density, rather than a feature of any system-specific biology. For example, one could modify our model of breeding migrations, in which individuals travel one way to discrete fixed end-points to breed, to fit many species of migratory birds, by adding another equivalent movement stage reflecting the navigational challenges on their return journey back to the feeding grounds. Alternately, one could assume that our navigational accuracy term, *a*(*U*), applies, as is, to the round trip to the feeding grounds and then back to the breeding grounds and simply remove the assumption of semelparity.

Migratory populations may face a multitude of external perturbations to their survival. In our models we have accounted for such processes by implementing a generic mortality term, *h*. This term represents effects such as additional mortality due to harvesting, the introduction of new diseases or predators, or climate change. It could also represent impedances to migration such as dams, roads, buildings or reduction of migration corridors, which in addition to causing mortality, may restrict the ability, or tendency, of animals to move.

We have focused on collective navigation as the main group benefit during migrations, and include this benefit as a monotonic increase in accuracy (breeding migrations) or resource tracking ability (feeding migrations) as a function of population size (Eq. (3)). One could, in principle, recast the *a*(*U*) term as a general benefit of collective behavior, such as an increase in probability to properly time a migration [64–66] or survive predation en route, or a boost in efficiency due to aerodynamic benefits [67] or by collectively navigating a more efficient route [12].

We implicitly assume that greater population density results in larger typical group sizes. This is supported by empirical [68] and theoretical [9, 69] results that suggest that this is the case for social species. For simplicity, in the breeding migration model we assume that the population is limited by resources on the feeding grounds. Though not shown, we confirmed numerically that assuming that the limiting resource is on the breeding grounds does not change our results. Also for simplicity, in the feeding migrations we assume that the group tracks only a single favorable region, does so indefinitely and breeds continuously along the way. We stress that a population needs only to be limited by a dynamic resource field for a portion of their life-cycle (or the season) for this model to qualitatively apply.

Populations of migratory schooling fishes, including striped bass, capelin, herring, sardine, anchovy and cod, subject to intense fishing pressure have collapsed and may be slow to recover (see [70] and references therein). Though there are other explanations for such collapses [71], this is consistent with the Allee effect predicted by our models. Similarly, caribou herds have ceased to migrate after population declines and only started again once the population recovered [72]. Associations between numbers of migrants and migration distance have been observed in wildebeest [73] and there are many historical examples of migration collapse for both the blue and black wildebeest [74], however little is known about the exact nature of these events.

More subtly, our multi-site model (Model 2) suggests that there may be critical levels of additional mortality at which local adaptation and population genetic structure collapses (Fig. 4). Our model did not explicitly consider interbreeding between locally adapted and non-locally adapted types, which could further erode local adaptation at a site, so the collapse we observe should perhaps be considered an upper bound on local adaptation. Feedbacks between local adaptation and dispersal may strengthen such a collapse [75]. This may be particularly relevant when we consider anadromous salmon, which do home more accurately in years of greater density [11, 30] and are locally adapted to their natal streams [48–50], however, they do not appear to suffer Allee effects when looking at population size [76, 77]. Though it might not be observable to stock managers with their eye on population enumeration and straying rates, this sudden shift in percentage of locally adapted fish could erode portfolio effects [78] which play an important role in stabilizing populations on larger scales [79].

## Conclusions

The models we develop in this paper show that if animals rely on collective navigation, we expect their population dynamics to exhibit Allee effects that introduce a critical population size below which the population collapses. We observe this in models of breeding as well as of feeding migrations. Regarding breeding migrations, when animals use collective navigation to find the breeding site for which they are locally adapted, population genetic structure can collapse without the total population size showing obvious signs of decline. In our model of feeding migrations, population collapse is accompanied by a cessation of migration, and there are two alternative stable states of the population: high density and migratory, or low density and sedentary. Analogous to the evolutionary results of [9], both the breeding and feeding models exhibit hysteresis, meaning that if a population’s size, genetic structure, or migratory state collapses due to a perturbation, simply removing that perturbation is not enough to recover the population’s previous state.

The results from our study highlight ecological and conservation implications of collective behavior. We point to the need for more in-depth studies testing the predictions from these models and in particular for looking into the mechanisms underlying such large-scale processes. Looking to the future, advances in automated video tracking and technologies such as unmanned aerial vehicles are going to yield better data to understand how animals interact with one other and with cues in the environment, especially during migrations. As noted by several authors, there remains a disconnect between mechanism-focused studies of (collective) behavior and ecosystem dynamics [80, 81]. We hope this work serves as a further step in linking group-level processes to ecosystem-level functioning.

## Additional files

Additional file 1 Appendix A. *R*-*N* phase diagrams for single site breeding migration model. (PDF 203 kb)

Additional file 2 Animation 1. Low mortality, large starting population. Movie depicting simulation of the feeding migration model (Model 3 – Eqs. (10)–(13)). The dynamic green curve shows the carrying capacity at all points in the 1D space. The location and height of the red bar indicate the location and size of the migratory population, respectively. In this scenario, low mortality levels *h* allow persistence of a large population, though somewhat smaller than the carrying capacity, which ‘keeps up’ with the moving patch of good conditions. (AVI 194,979 kb)

Additional file 3 Animation 2. Low mortality, small starting population. Curves and symbols as in animation i. This scenario has the same conditions (low mortality as in animation i), however the initial population size is low and as a result the population is not able to keep track of the patch and grow. The traveling wave of the good patch still coincides with the location of the population at regular intervals, but the slight increase in population size is insufficient to regain the capacity for efficient migration of the group. Together with animation i this represents the bistability found in the model (left side of Fig. 5 of the main text). (AVI 286,114 kb)

Additional file 4 Animation 3. High mortality, large starting population. Curves and symbols as in animation i. In this scenario the high mortality suppresses the population size so severely that the group is unable to maintain its migration successfully and stagnates, despite starting with a large population. This scenario corresponds to the right side of Fig. 5 of the main text. (AVI 317,661 KB)
