# Supplementary material for: Collective behavior as a driver of critical transitions in migratory populations
Source: Mov Ecol. 2016 Jul 15;4:18. doi: 10.1186/s40462-016-0083-8 (PMC4946155; doi:10.1186/s40462-016-0083-8)
Supplement: Additional file 1 — Appendix A. R-N phase diagrams for single site breeding migration model. (PDF 203 kb) [file 40462_2016_83_MOESM1_ESM.pdf]

Appendices for:

Collective behavior as a driver of critical transitions in migratory populations

---

Appendix A. *R*-*N* phase diagrams for single site breeding migration model

In this supplementary information we illustrate the stability properties of the equilibria in the single-site migration model, illustrated in Fig. 1 of the main text. We numerically characterize the properties of the system solutions (equations (1) & (4) in the main text) in regions i, ii, and iv, by means of showing the basins of attractions for solutions in these parameter regions. In Fig. A.1 we indicate the h-values (vertical red lines) for which trajectories and basins of attraction were studied. These regions exemplify the qualitatively distinct stability properties that characterize the system over a range of values for parameter h.

The single-site system exhibits a single stable equilibrium in region ii (Fig. A.1), i.e. for low values of additional mortality ($h \lesssim 0.26$), the population always persists with positive densities and the zero equilibrium is unstable, see Fig. A.2(a). For higher mortality levels the system has a bistable region, in which in addition to the stable equilibrium with positive population numbers (solid blue dot), the zero equilibrium is also stable (solid red dot), while an unstable equilibrium (empty black circle) separates the two stable equilibria from one another (Fig. A.1, Fig. A.2(b)). Over a small range of h-values in region i, the system dynamics are characterized by the occurrence of an unstable limit cycle (dashed black loop), which originates from the subcritical Hopf bifurcation, separating regions i and iv (Fig. A.2(c)). The limit-cycle amplitude increases towards lower h-values, until the cycle coincides with the stable manifold of the unstable equilibrium (saddle node) and the cycle disappears through a homoclinic cycle (global bifurcation). The Hopf bifurcation demarcates the h-value for which the equilibrium with positive population numbers becomes unstable and for higher mortality levels, only the zero-equilibrium is stable (Fig. A.2(d)).

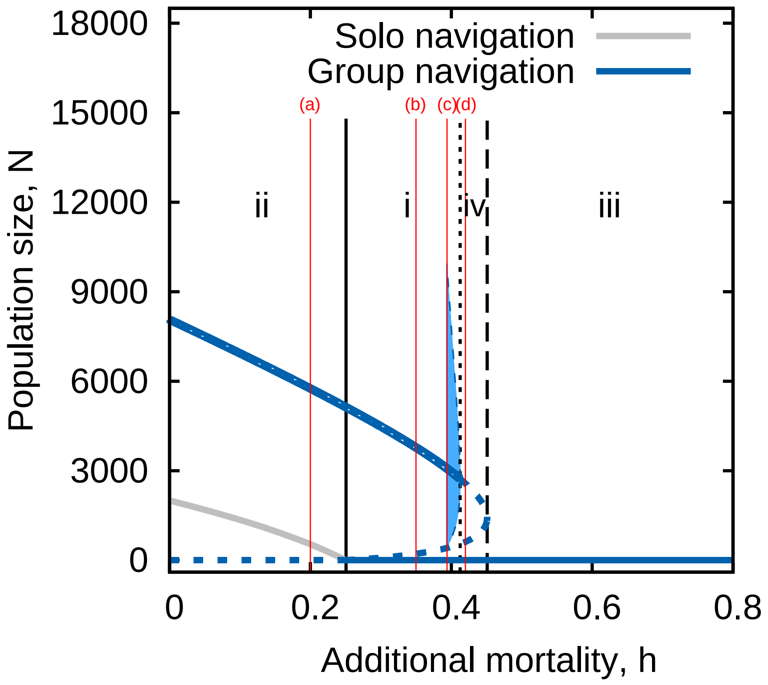

Figure A.1: Main text Fig. 1b is redrawn here. The red vertical lines indicate the *h*-values for which we show the *R*-*N* state-space analysis and basins of attraction for system solutions in Fig. A.2. The panel labeling of Fig. A.2 is shown above the red lines.

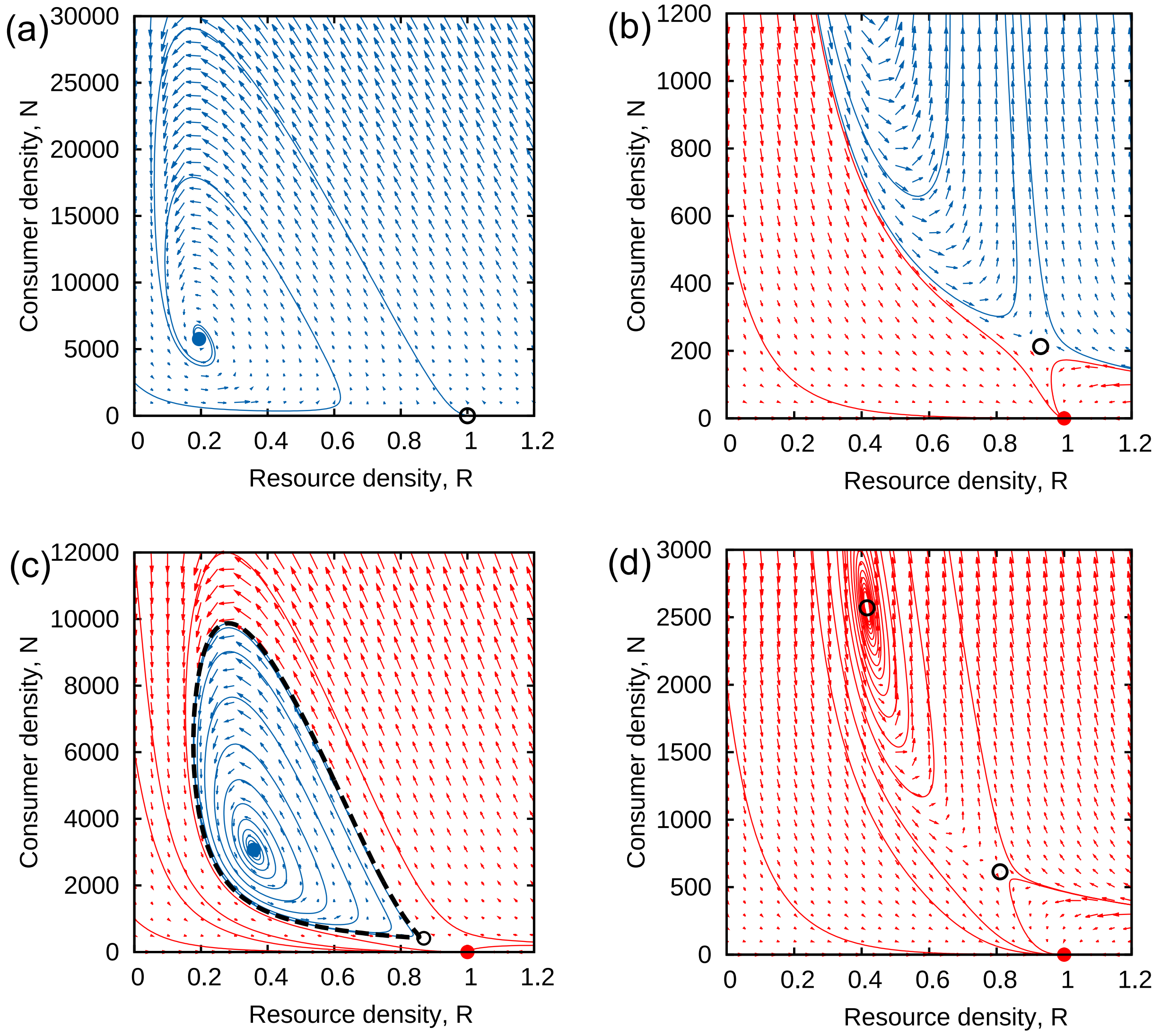

Figure A.2: State-space analysis of the single-site system (equations (1) & (4) in the main text) depicted for the system variables R and N. We show the basins of attraction for system solutions in three regions of parameter space, with mortality levels as indicated in Fig. A.1 ($h = 0.20$, $h = 0.35$, $h = 0.394$ and $h = 0.42$ for panels (a), (b), (c) and (d), respectively). Stable equilibria are indicated with solid circles, unstable equilibria with open circles, and the unstable limit cycle is indicated with a dashed line. Trajectories through state space are shown with thin solid lines and their color corresponds to the equilibrium to which they lead. Arrows depict the local gradient in state space, and similarly their colors correspond to the basins of attraction of the stable equilibria. Panel (a) shows the global basin of attraction for the stable equilibrium with positive population size in region ii. In panel (b) the bistability in region i is shown, including the separation of the basins of attraction of the two stable equilibria (here, the stable equilibrium with non-zero population size is outside of the range shown). For a small range of h-values in region i we find an unstable limit cycle, shown in panel (c). Trajectories that are initiated within the unstable limit cycle's basin of attraction approach the stable equilibrium with positive population densities (blue trajectories to blue solid circle). This means that this equilibrium is only locally stable. When trajectories are initiated far from that stable equilibrium, i.e. outside the unstable limit cycle, the system approaches the zero equilibrium (red trajectories and red solid circle). Even higher mortality values, in region iv, do not allow for persistence and the zero equilibrium is the only stable solution in the system, as shown in panel (d).
